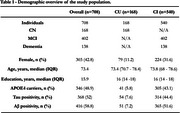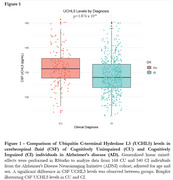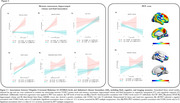# The Association of UCHL5 Levels with AT(N) Biomarkers

**DOI:** 10.1002/alz70855_105205

**Published:** 2025-12-24

**Authors:** Sofia Gallo Salvadori, Gabriela Mantovani Baldasso, Christian Limberger, Eduardo R. Zimmer

**Affiliations:** ^1^ Universidade Federal do Rio Grande do Sul, Porto Alegre, Rio Grande do Sul, Brazil; ^2^ Universidade Federal do Rio Grande do Sul, Porto Alegre, RS, Brazil; ^3^ McGill University, Montreal, QC, Canada; ^4^ Brain Institute of Rio Grande do Sul ‐ Pontifícia Universidade Católica do Rio Grande do Sul, Porto Alegre, Rio Grande do Sul, Brazil

## Abstract

**Background:**

Alzheimer's disease (AD) is a proteinopathy characterized by the accumulation of amyloid‐β (Aβ) and hyperphosphorylated tau, leading to the formation of amyloid plaques and neurofibrillary tangles. Dysfunction of the ubiquitin‐proteasome system has been implicated in these pathological processes. Ubiquitin C‐terminal hydrolase L5 (UCHL5), a deubiquitinase that regulates proteasomal activity, plays a crucial role in degrading damaged proteins. However, its role in AD progression remains poorly understood. This study aims to investigate the association between UCHL5 levels in cerebrospinal fluid (CSF) and AD biomarkers.

**Method:**

We assessed 168 cognitively unimpaired (CU) and 540 cognitively impaired (CI) individuals from ADNI (Table 1), with available baseline FDG‐PET, Aβ‐PET, cognitive assessments, CSF and plasma biomarkers. The association between CSF UCHL5 levels and CSF Aβ42, total tau, *p*‐Tau181, plasma GFAP, hippocampal volume, Mini‐Mental State Exam (MMSE), Aβ‐, and FDG‐PET were analyzed using generalized linear mixed models adjusted for age and sex. Additionally, voxel‐wise analyses explored the association of UCHL5 levels with FDG‐ and Aβ‐PET.

**Result:**

Our analysis revealed higher UCHL5 CSF levels in CU compared to CI individuals (Figure 1). In CU, UCHL5 CSF levels were positively associated with *p*‐Tau181 and total tau levels (Figures. 2A, 2B). Additionally, CI individuals showed positive correlations between UCHL5 CSF levels and CSF Aβ42 ratio, plasma GFAP, MMSE scores, and hippocampal volume (Figures. 2C, 2D, 2E and 2F). Notably, UCHL5 CSF levels were negatively associated with AV45 SUVR rates of Aβ burden, in occipital, temporal and predominantly in parietal and frontal cortices (Figure 2G). On the other hand, a positive correlation was observed between UCHL5 CSF levels and FDG SUVR values, further supported by FDG‐PET voxel‐wise analyses, highlighting prefrontal cortex, temporal gyrus and parietal lobe (Figure 2H).

**Conclusion:**

These findings reveal intriguing correlations between AT(N) biomarkers and CSF UCHL5 levels, suggesting a potential role for this protein across different stages of Alzheimer's disease. However, further analyses are needed to clarify UCHL5's involvement in early tau accumulation, clinical conversion, and the degradation of misfolded proteins associated with AD proteinopathies, as well as its impact on neurodegeneration.